# Impact of Bradykinin Generation During Thrombolysis in Ischemic Stroke

**DOI:** 10.3389/fmed.2018.00195

**Published:** 2018-07-03

**Authors:** Maxime Gauberti, Fanny Potzeha, Denis Vivien, Sara Martinez de Lizarrondo

**Affiliations:** ^1^Normandie Univ, UNICAEN, Institut National de la Santé et de la Recherche Médicale UMR-S U1237, “Physiopathology and Imaging of Neurological Disorders” PhIND, Caen, France; ^2^Department of Diagnostic Imaging and Interventional Radiology, Centre Hospitalier Universitaire Caen Côte de Nacre, Caen, France; ^3^Department of Clinical Research, Centre Hospitalier Universitaire Caen, Caen, France

**Keywords:** contact phase, fibrinolysis, angioedema, inflammation, brain edema, blood-brain barrier, factor XII, kininogen

## Abstract

Ischemic stroke is one of the leading causes of death and disability worldwide. Current medical management in the acute phase is based on the activation of the fibrinolytic cascade by intravenous injection of a plasminogen activator (such as tissue-type plasminogen activator, tPA) that promotes restauration of the cerebral blood flow and improves stroke outcome. Unfortunately, the use of tPA is associated with deleterious effects such as hemorrhagic transformation, symptomatic brain edema, and angioedema, which limit the efficacy of this therapeutic strategy. Preclinical and clinical evidence suggests that intravenous thrombolysis generates large amounts of bradykinin, a peptide with potent pro-inflammatory, and pro-edematous effects. This tPA-triggered generation of bradykinin could participate in the deleterious effects of thrombolysis and is a potential target to improve neurological outcome in tPA-treated patients. The present review aims at summarizing current evidence linking thrombolysis, bradykinin generation, and neurovascular damage.

## Introduction

Stroke represents the second leading cause of mortality in the world, the third leading cause of reduced disability-adjusted life years and is a major drain on public health-care funding (1). Most strokes are ischemic in nature (80%) and occur when an artery that supplies blood to the brain is blocked by a blood clot (2, 3). The aim of the initial medical management is to limit the extension of brain damage by recanalizing the occluded artery (4). To date, thrombolysis (injection of tissue-type plasminogen activator (tPA), sometimes combined with endovascular thrombectomy), is the only pharmacological treatment for acute ischemic stroke (5). It acts by activating an inactive zymogen, plasminogen, into its active form, plasmin, which is itself able to degrade fibrin, the main constituent of blood clots (6).

In clinical trials, intravenous tPA injection has been shown to improve neurological outcome when injected up to 4.5 h after the appearance of symptoms (7). Yet, tPA administration is not devoid of side effects (6, 8). For instance, when tPA is administered after 6 h, meta-analysis of clinical trials showed that it worsens the neurological outcome (9). Moreover, even when injected in the right therapeutic window, current evidence supports that tPA increases by up to 60% the mortality of ischemic stroke patients in the first 7 days (8). This increased mortality is partly explained by an increased rate of hemorrhagic transformation. It has also been demonstrated that stroke patients treated with tPA present more severe brain edema, with malignant edema being 2.7 fold more frequent after tPA administration in patients with occlusion of the internal carotid artery (10). These data suggest that besides its beneficial effects on the cerebral blood flow, tPA administration also exerts deleterious effects on the neurovascular unit (11). Therefore, blockade of these deleterious effects may significantly improve the clinical efficacy of tPA mediated thrombolysis and increase its therapeutic window. However, to date, the mechanisms driving the deleterious effects of tPA remain poorly understood and probably include both plasmin-dependent (12) and plasmin-independent processes (11).

There is now compelling evidence supporting that tPA-induced bradykinin generation could be one of the drivers of the deleterious effects of tPA (13–15). Bradykinin is a potent pro-inflammatory and pro-edematous peptide, that is generated by proteolytic cleavage from its precursor high-molecular weight kininogen (HMWK) (16, 17). HMWK cleavage is classically triggered by the activation of the contact system, which is also composed of factor XII (FXII), factor XI (FXI), and plasma prekallikrein (PPK). The contact system is started by FXII binding to negatively charged surfaces such as platelet polyphosphate and mast cell heparin (18). Notably, there is a significant generation of bradykinin during thrombolysis in stroke patients (14, 19). Given the multiple roles for bradykinin in the neurovascular unit described so far including increase in BBB permeability, inflammatory cytokines upregulation, release of glutamate by astrocytes and microglial activation (all potential pathogenic players in ischemic stroke) (17), it is tempting to speculate that the generation of bradykinin triggered by tPA administration participates in its deleterious effects.

The aim of this present review is to summarize current evidence linking tPA administration, bradykinin generation, and neurovascular damage in stroke patients. In the first part of this review, we will present clinical evidence supporting the existence of deleterious effects of tPA on the neurovascular unit. Then, we will describe the molecular pathways linking tPA administration to bradykinin generation. Finally, we will recapitulate the most recent preclinical evidence on the effects of bradykinin in the ischemic brain.

## The effects of tPA on the neurovascular unit: clinical evidence

Intravenous thrombolysis is now an established stroke treatment for patients presenting within 4.5 h after stroke onset (20). Indeed, large, multicenter, randomized, and controlled clinical trials demonstrated that intravenous tPA administration improves stroke outcome in this subset of patients (7). Although tPA is beneficial for most patients, there is a growing body of evidence that tPA may also harm, in particular when tPA is administered more than 6 h after symptom onset. It is therefore tempting to speculate that the outcome after tPA administration in stroke patients depends on a balance between its beneficial and deleterious effects. Exhaustive literature reviews have been published on the preclinical effects of tPA on the neurovascular unit (6, 11, 21). Here, we will focus only on clinical evidence and extract the data that are relevant to the understanding of the pathophysiology of stroke in the context of tPA administration.

### Beneficial effects

Regarding the beneficial effects of tPA, they appear mainly dependent on its ability to promote arterial recanalization. For instance, in patients presenting occlusion of the termination of the internal carotid artery, in which the rate of recanalization is estimated to be <5% (22), the ICARO study (case-control study) demonstrated that there is no beneficial effect of tPA administration vs. placebo (10). Additionally, patients who recanalize after tPA administration have a far better outcome than patients without recanalization as demonstrated by a large number of studies (23). However, tPA seems also to benefit patients without visible arterial occlusion at presentation (24). Although this can be explained by the presence of distal microemboli not seen on angiography, we cannot exclude the existence of beneficial effects of tPA that are independent of its ability to promote arterial recanalization (25). These putative fibrinolysis-independent effects are relevant for the design of adjuvant treatments for tPA, since these treatments should aim at mitigating the deleterious effects of tPA without jeopardizing its beneficial effects.

### Symptomatic intracranial hemorrhage

The main deleterious effects of tPA highlighted by clinical studies are symptomatic intracranial hemorrhage (sICH), symptomatic brain edema and angioedema. sICH is probably the most feared complication of tPA administration with a mortality rate approaching 50% (26). The risk of sICH after tPA administration ranges from 2 to 7% according to the definition used. Most of them occur within 24 h after tPA administration with a median time ranging from 5 to 10 h. sICH are related to the dose of tPA administered, as recently demonstrated in a randomized study comparing the standard dose of tPA (0.9 mg/kg) to a lower dose (0.6 mg/kg) that showed sICH rates of 2.1% vs. 1.0% respectively (27). In contrast, time from symptom onset to tPA administration is not significantly associated with sICH risk according to a large meta-analysis (28), although there is a trend for a higher risk when tPA administration is delayed (7, 9). Interestingly, an increased risk of sICH has also been demonstrated after administration of other plasminogen activators such as streptokinase or urokinase (29, 30). Since these plasminogen activators are structurally unrelated, this suggests that the pro-hemorrhagic effect of tPA is dependent on plasminogen activation. Reperfusion and subsequent reperfusion-injury do not seem to explain alone the pro-hemorrhagic effect of tPA since hemorrhage can occur in areas remote from the infarcted tissue (31) and in the absence of reperfusion (32). Given that human imaging studies demonstrated that breakdown of the blood brain barrier (BBB) precedes sICH (33), these data altogether suggest that tPA administration leads to BBB breakdown and subsequent sICH by a plasminogen dependent mechanism. As a concomitant phenomenon, tPA administration induces a coagulopathy characterized by fibrinogen depletion and prolongation of prothrombin and partial thromboplastin times which may also increase the risk of sICH (34, 35).

### Symptomatic brain edema

Symptomatic brain edema is a less known complication of tPA treatment, although its effect on mortality is significant (36). In the IST-3 trial for instance, 3% of tPA-treated patients (47/1515) died from symptomatic swelling of the ischemic lesion. Symptomatic brain edema is usually defined as a severe brain swelling with mass effect on imaging. The independent effects of tPA on brain edema are difficult to measure since the reperfusion achieved using tPA is associated with a reduced ischemic lesion size and therefore, a reduced brain swelling. In this regard, the case-control ICARO study that involved stroke patients with occlusion of the internal carotid artery is interesting, since the recanalization rate is expected to be <5% in this subset of patients, allowing to observe the effects of tPA almost independently of arterial recanalization (10). In this study involving 253 patients per group, the rate of fatal symptomatic brain edema (referred as malignant edema) was 8.3% in tPA-treated patients vs. 3.1% in controls (*p* = 0.013). As a comparison, the rate of fatal sICH was only 2.8% in tPA-treated patients, suggesting that the contribution of symptomatic brain edema to mortality is larger than sICH in this patient subset. In a cohort of 943 tPA-treated patients, Strbian et al. studied the parameters associated with the development of brain edema (36). They found imaging evidence of brain edema in 28% of tPA-treated patients and severe forms in 10%. The factors associated with edema development were higher baseline National Institutes of Health Stroke Scale, presence of hyperdense cerebral artery sign or early infarct signs, and longer treatment delays. Altogether, these data suggest that tPA administration promotes brain edema, especially in patients showing early sign of brain infarction on imaging and experiencing longer onset-to-tPA delays. Whether this pro-edema effect is dependent on plasminogen activation cannot be easily retrieved from clinical data.

### Angioedema

Angioedema is a rare complication of tPA administration, occurring in ~2% of patients with ischemic stroke (37). It manifests usually as a hemi-orolingual edema during or shortly after tPA administration that can be life threatening. Angioedema is associated with the use of angiotensin-converting enzyme (ACE) inhibitors, which is found in 45% of patients with angioedema (19). This finding unraveled bradykinin as the causative factor for tPA-induced angioedema. Indeed, ACE is not only able to process angiotensin into angiotensin-II, it is also responsible for the degradation of bradykinin. Thus, the plasma half-life of bradykinin is extended in patients taking ACE inhibitors, thereby increasing the plasmatic concentration in case of bradykinin generation, that can more easily reach a level triggering angioedema (38). Although the occurrence of angioedema does not significantly influence neurological outcome, patients with angioedema tend to have more sICH and more malignant brain edema according to the largest series published so far (37). Overall, these clinical data support the following mechanism: tPA administration triggers bradykinin generation, which is itself able to promote angioedema, especially in patients taking ACE inhibitors. In line with the tendency for a higher incidence of sICH and malignant brain edema in patients presenting angioedema, it is tempting to speculate that the effects of tPA-induced bradykinin generation are not restricted to the occurrence of angioedema but also influence the evolution of the ischemic lesion in the brain.

## Molecular mechanisms of bradykinin generation after tPA administration

The first convincing evidence of bradykinin generation after tPA administration was published by Agostoni et al. They showed that intravenous administration of tPA or streptokinase in patients with acute myocardial infarction was associated with a cleavage of HMWK, the precursor of bradykinin (13). Interestingly, more HMWK was cleaved after streptokinase than after tPA administration, pointing toward plasmin as a key player in this mechanism, since it has been demonstrated that streptokinase generates more plasmin than tPA after injection (39). Later, the same results were obtained in ischemic stroke patients treated with tPA (14). To be able to interfere with the generation of bradykinin after tPA administration, it is therefore relevant to understand first how tPA generates plasmin and then, how plasmin generates bradykinin (40).

### tPA-induced plasmin generation

tPA cleaves plasminogen at its Arg561—Val562 peptide bond, into the active fibrinolytic enzyme, plasmin (41). Although tPA can activate plasminogen into plasmin in the plasma, its efficiency is 100 times higher in the presence of fibrin as a cofactor (42). This role for fibrin as a cofactor for plasminogen activation explains the relative fibrin specificity of tPA compared to streptokinase. This provides a mechanistic explanation for the above mentioned higher systemic generation of plasmin when streptokinase is used as a fibrinolytic agent. Moreover, this implies that, at the level of the thrombus which contains large amounts of fibrin, there is a massive generation of plasmin and therefore, a potentially intense generation of bradykinin. Accordingly, this locally generated bradykinin could act on the ischemic brain downstream of the occlusion site.

### Plasmin-induced bradykinin generation

The mechanism by which plasmin generates bradykinin is debated. The first described mechanism is based on the fact that plasmin is able to directly cleave HMWK and generates bradykinin *in vitro* (14, 19). This has been demonstrated using electrophoresis of HMWK after incubation with tPA, plasminogen, tPA plus plasminogen and plasmin. Whereas no cleavage occurred in the presence of tPA or plasminogen when added alone, a significant cleavage of HMWK occurred in the presence of plasmin or tPA plus plasminogen. This cleavage was accompanied with bradykinin release as assessed by quantification of immunoreactive bradykinin. Although this direct mechanism is apparent *in vitro*, whether it occurs *in vivo* and participates in bradykinin generation after intravenous injection of tPA remains unknown.

An alternative, indirect mechanism of bradykinin generation after plasmin formation has been described by Simão et al. They first observed that the incubation of human plasma with tPA leads to the formation of active plasma kallikrein (PKal), the enzyme of the contact phase responsible for “physiologic” HMWK cleavage (43). Interestingly, the increase in PKal activity after addition of tPA was lost in Factor XII (FXII) and plasminogen deficient plasma (15). A set of experiments allowed to dissect the complete cascade: tPA activates plasminogen into plasmin, plasmin activates FXII into FXIIa and finally, FXIIa activates plasma prekallikrein into PKal. In line with these *in vitro* findings, intravenous injection of tPA in mice leads to an increase in PKal activity. As expected, this increased PKal activity leads to a cleavage of HMWK. This indirect mechanism of bradykinin formation is probably the dominant mechanism after tPA administration, since tPA does not induce HMWK cleavage in FXII or plasma prekallikrein deficient mice, even if there is plasmin generation in these animals.

**Figure 1:**
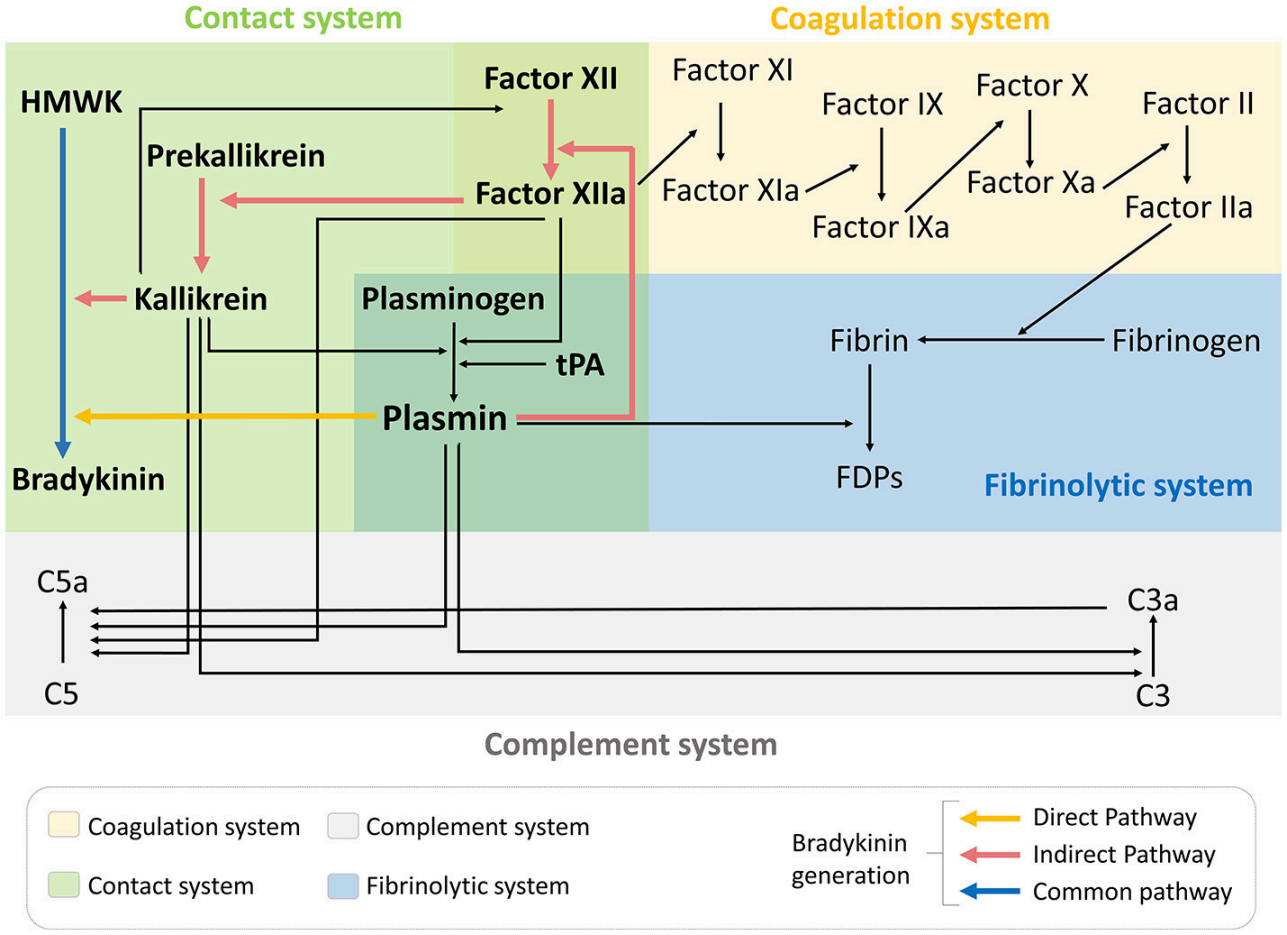
tPA administration drives hemovascular dysfunction. Thrombolytic therapy in the acute phase of ischemic stroke promotes activation not only of the fibrinolytic pathway but also coagulation, complement and contact phase systems. FDPs, fibrin degradation products; HMWK, high molecular weight kininogen; tPA, tissue-type plasminogen activator.

Besides activation of the contact phase and subsequent bradykinin generation, there is also a growing body of evidence suggesting that tPA administration induces a widespread hemovascular dysfunction also involving activations of the coagulation cascade and the complement system (Figure 1) (44–46). All these biochemical cascades can participate in the deleterious effects of tPA on the neurovascular unit in the acute stroke settings.

## Bradykinin generation as a unifying mechanism for the effects of tPA on the neurovascular unit

Bradykinin is a nonapeptide (Arg^1^-Pro^2^-Pro^3^-Gly^4^-Phe^5^-Ser^6^-Pro^7^-Phe^8^-Arg^9^) released from HMWK after its cleavage. Bradykinin acts through two receptors: kinin 1 receptor (B1R) and kinin 2 receptor (B2R). It requires further processing to Des-Arg^9^-Bradykinin by peptidases (such as carboxypeptidase N) to act through B1R. B2R is almost ubiquitously expressed in the brain (including in the cerebral cortex) whereas B1R is inducible and overexpressed in pathological conditions. B1R and B2R have been described on all the main cellular cell types of the brain including neurons, astrocytes, microglia, oligodendrocytes, and endothelial cells (47). Interestingly, both receptors are upregulated in the ischemic brain (17). Thus, it has been demonstrated that B1R and B2R are significantly overexpressed at the protein level as early as 4 h post-stroke onset in mice (48). It is therefore tempting to speculate that the effects of tPA-induced bradykinin generation on the neurovascular unit are time-dependent with a larger effect size when tPA administration is delayed. B2R activation has also been shown to induce tPA release from endothelial cells, potentially further amplifying bradykinin generation (49).

### Contradictory findings regarding bradykinin impact in stroke

**Figure 2:**
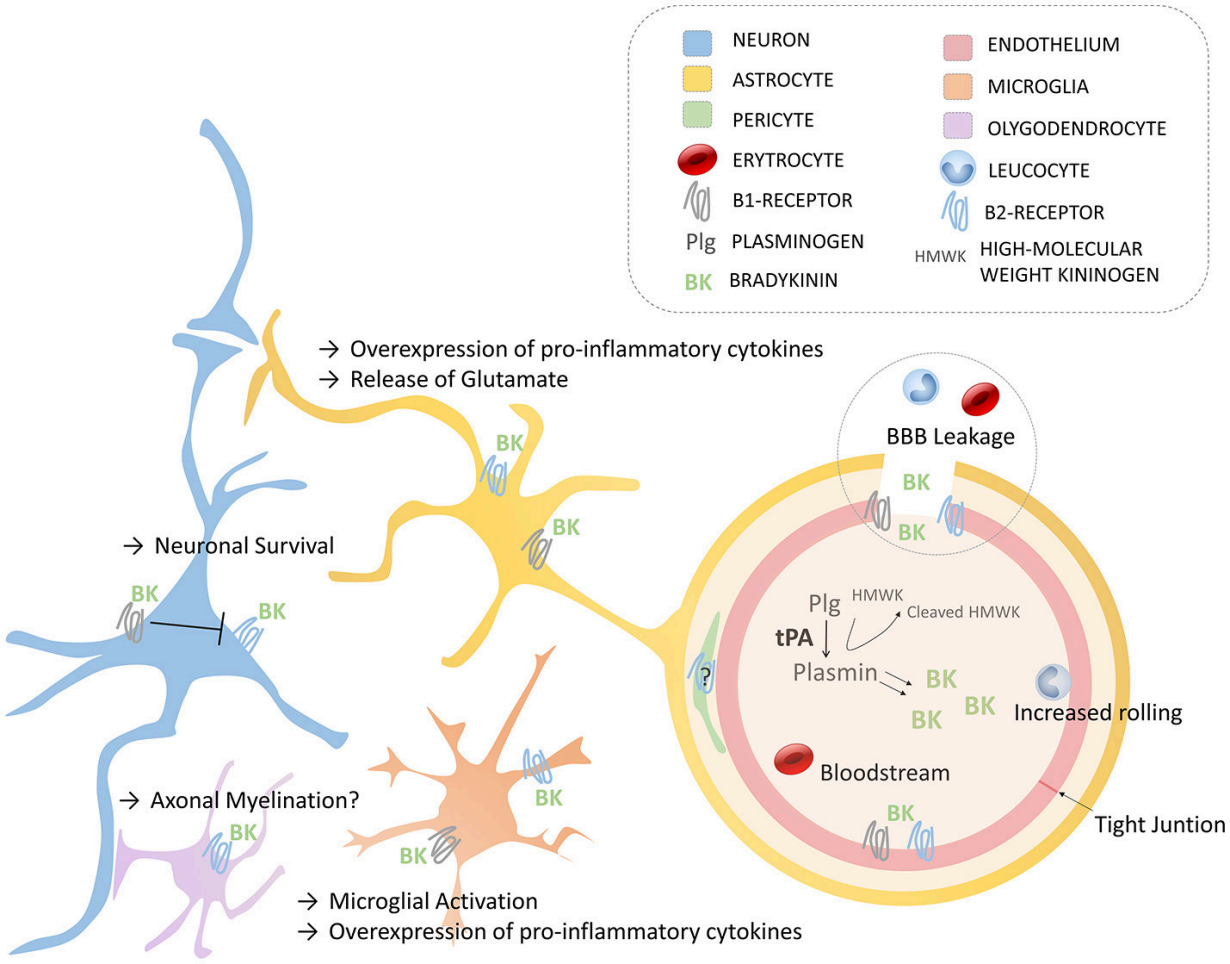
Schematic of the main roles of bradykinin in the Neurovascular Unit via B1- and B2- Receptors. Endothelium (with tight junctions), astrocytes, neurons, oligodendrocytes, microglia, and pericytes compose the neurovascular unit. BBB, blood-brain barrier; BK, bradykinin; HMWK, High molecular Weight Kininogen; Plg, plasminogen; tPA, tissue-type plasminogen activator.

The implication of the kallikrein-kinin system in neurological disorders has been recently reviewed (17). Here, we will focus mostly on the effects of bradykinin relevant to thrombolysis and stroke. The effects of endogenous bradykinin on the ischemic brain are numerous and the results of experimental studies are often contradictory (47). For instance, genetic deletion or pharmacological blockade of B2R has been shown to protect from stroke in a number of studies, mainly performed using the transient mechanical vascular occlusion model of stroke (filament) (50–52). In contrast, some studies reported the absence or even deleterious effects of B2R inhibition using similar experimental models (48, 53). In addition, intravenous injection or overexpression of tissue kallikrein (able to generate kallidin, a bradykinin analog, after cleavage of low-molecular kininogen) has beneficial effects in stroke, which are dependent on B2R activation (54, 55). Regarding B1R, the data are more homogenous since most studies described deleterious effects on ischemic lesion size, brain edema and post-stroke inflammation (48, 56–58). It is however difficult to isolate the contribution of bradykinin on each of these pathophysiological processes, since reduction of lesion size itself is associated with reduced brain edema and inflammation. In this context and in the absence of experimental studies investigating the effects of bradykinin receptor blockade after tPA administration, we can only make hypotheses regarding the impact of bradykinin in the deleterious effects of tPA (Figure 2), as summarized thereafter.

### Bradykinin enhances BBB permeability

The most documented effect of bradykinin on the neurovascular unit is an increase in the permeability of the BBB. From a mechanistical point of view, bradykinin binding on either B1R or B2R promotes intracellular calcium release, leading to downregulation of claudin-5 (one of the main mediators of BBB integrity) and subsequent BBB leakage (17). Accordingly, B2R agonists (and to a lesser extent, B1R agonists) have been used as a mean to transiently increase BBB permeability for maximizing brain bioavailability of chemotherapeutic drugs (59, 60). In the context of stroke, this bradykinin-induced BBB leakage is considered deleterious since it allows macromolecules to reach the brain parenchyma, thereby promoting brain edema, and weakens the BBB, thereby increasing the risk of hemorrhagic transformation (61). In line with these hypotheses, in mice with thrombotic occlusion of the middle cerebral artery, tPA administration increases hemorrhage transformation, infarct volume and edema (15). Of note, these deleterious effects of tPA are prevented by a PKal inhibitor, pointing toward contact phase activation (and possibly bradykinin generation) as the main pathophysiological player. This might explain why tPA-treated patients with angioedema, who supposedly generate more bradykinin, tend to have more sICH and more malignant brain edema (37). When tPA administration is delayed, the increased expression of bradykinin receptors by the ischemic brain might also explain the higher rate of symptomatic brain edema and the trend for a higher risk of hemorrhagic transformation (9, 36).

### Pro-inflammatory effects of bradykinin

Beside increasing BBB permeability, bradykinin displays pro-inflammatory effects in the cerebral circulation (17). For instance, direct intra-carotid injection of bradykinin in gerbils induces an increased rolling of leukocytes and adhesion of platelets on the brain endothelium, an effect dependent on B2R (62). The mechanisms by which bradykinin mediates post-stroke inflammation is classically described as involving the activation of B2R (on endothelial cells, astrocytes, neurons, microglia), the release of arachidonic acid and the activation of cyclo-oxygenase (COX) enzymes (63–65). Bradykinin is also a potent stimulator of the expression of other inflammatory mediators such as cytokines and acts as a leucocyte chemoattractant (66). More recently, inhibition of B1R (but not B2R) has been shown to reduce post-ischemic inflammation in a transient middle cerebral artery occlusion model, in terms of proinflammatory cytokines expression and extent of brain invasion by immune cells (48), but these results can be biased by the dramatic reduction of ischemic lesion size in case of B1R inhibition. Overall, it is reasonable to assume that bradykinin has pro-inflammatory effects through both B1R and B2R activations (17). Bradykinin may also activate mast cells, that play a significant role in tPA-induced brain edema and hemorrhagic transformation (67, 68). All these bradykinin-mediated mechanisms could, at the very least, participate to the pro-inflammatory effects of tPA, as observed in several experimental studies (69). However, brain inflammation after stroke is a dual-edged sword, with inflammatory cascades stimulating deleterious but also potentially beneficial pathways, including post-injury repair processes (70). Thus, the net effect of additional bradykinin generation triggered by tPA-administration in the context of acute ischemic stroke is difficult to predict and could explain the Janus-faced nature of tPA in this context (6).

### Bradykinin and neuronal cell death

Bradykinin can also directly influence neuronal cell fate in the ischemic brain, as recently reviewed (71). In particular, B2R protects against neuronal cell death via multiple molecular mechanisms including anti-apoptotic, anti-oxidative, anti-inflammatory, anti-autophagic, and anti-excitotoxic effects (excitotoxicity refers to the damage triggered by excessive stimulation of neurons by neurotransmitters). Bradykinin displays also a B2R dependent vasodilatory effect on brain microvessels that could improve cerebral perfusion and indirectly mitigate neuronal cell death (63). Thus, bradykinin-mediated B2R activation may participate in the beneficial effects of tPA administration in patients without visible arterial occlusion (24). *In vitro* experiments suggest that there is a crosstalk between B1R and B2R during pro-excitotoxic challenges in neurons (72). It has been reported that bradykinin triggers a neuroprotective cascade via B2R activation in hippocampal neurons conferring protection against excitotoxicity. Notably, co-activation of B1R blocks this protective effect. Since carboxypeptidases allowing bradykinin processing and subsequent B1R activation are highly expressed in the brain (47), this suggests that the beneficial effects of B2R activation in stroke might be blocked by concomitant B1R activation after bradykinin processing. This suggests that B1R or carboxypeptidase blockade could promote neuroprotection, especially in the context of thrombolysis for acute ischemic stroke. Since B1R expression is induced by brain ischemia, this may also explain why the beneficial effects of tPA administration are larger when thrombolysis is performed in the first hours following stroke onset (9).

## Conclusion and therapeutic perspectives

Even if there are probably several molecular effectors of the deleterious effects of tPA on the neurovascular unit in acute ischemic stroke, current pathophysiological knowledge suggests that bradykinin could be one of the most important players. In particular, the pro-inflammatory and pro-edematous effects of bradykinin could explain the increased incidence of sICH and symptomatic edema in tPA-treated patients. If true, angioedema would be only the tip of the iceberg of bradykinin effects in tPA-treated stroke patients. In line with this hypothesis, inhibition of bradykinin receptors or bradykinin processing peptidases could improve the efficacy of tPA and stroke outcome. The challenge resides in the adequate choice of the inhibitors in order to block the deleterious effects without jeopardizing the beneficial effects of bradykinin. The repercussion of such strategy could be large not only in acute ischemic stroke but also in other diseases where pharmacological thrombolysis is indicated including intraventricular brain hemorrhage, acute myocardial infarction, pulmonary embolism, and limb ischemia. This should also stimulate the development of new thrombolytic strategies that act independently of plasmin generation, such as a disintegrin and metalloprotease with thrombospondin type I repeats-13 (ADAMTS-13) or N-Acetylcysteine (73, 74).

## Author contributions

All authors listed have made a substantial, direct and intellectual contribution to the work, and approved it for publication.

### Conflict of interest statement

The authors declare that the research was conducted in the absence of any commercial or financial relationships that could be construed as a potential conflict of interest.
